# ST-elevation in aVR with Diffuse ST-segment Depression: Need for Urgent Catheterization?

**DOI:** 10.5811/cpcem.35284

**Published:** 2024-12-17

**Authors:** Bruce M. Lo, Megyn K. Christensen, Katherine E. Schaffer, Theodore J. Tzavaras

**Affiliations:** * Sentara Norfolk General Hospital/Eastern Virginia Medical School at Old Dominion University, Norfolk, Virginia; † Eastern Virginia Medical School at Old Dominion University, Norfolk, Virginia

**Keywords:** acute coronary syndrome, aVR, ST-segment elevation

## Abstract

**Case Presentation:**

A 33-year-old female with a history of antiphospholipid syndrome presented with exertional chest pain and ST-elevation in aVR with diffuse ST-segment depression. An emergent catheterization was performed, which showed an isolated 99% stenosis in the left main coronary artery. The remaining coronary arteries were without any stenosis. Successful stent placement was performed, and the patient was discharged without complications.

**Discussion:**

Previous guidelines have suggested that ST-segment elevation with diffuse ST-segment depression should be treated as a ST-elevation myocardial infarction equivalent involving either the left-main or proximal left anterior descending coronary artery. However, recent data suggests that most of these cases may not involve that region. Regardless, this electrocardiogram finding should still be a concern for acute coronary syndrome, with the need for urgent catheterization.

## CASE PRESENTATION

**Image 1:**
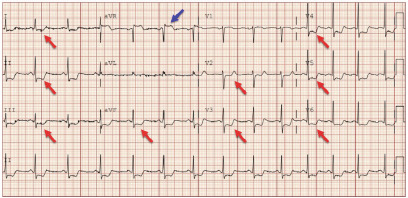
Electrocardiogram with ST-segment elevation in aVR (blue arrow) and diffuse ST-segment depression (red arrow).

**Image 2:**
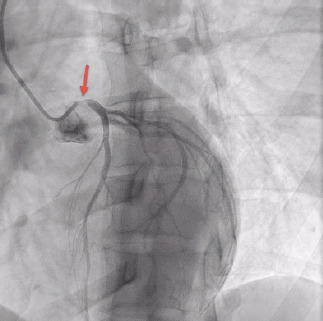
Cardiac catheterization showing 99% stenosis in the left main coronary artery (arrow).

A 33-year-old female with a history of antiphospholipid syndrome (APS), presented with exertional chest pain and shortness of breath. An electrocardiogram (ECG) was performed (Image 1), which showed ST-elevation in aVR with diffuse ST-depression. The patient was taken emergently for a heart catheterization, which showed a 99% stenosis at the ostial left main artery (Image 2). The remaining coronary arteries showed no disease. The patient had a drug-eluting stent placed with improvement to 0% stenosis, and she was discharged home several days later without event.

## DISCUSSION

ST-segment elevation in aVR with diffuse ST-segment depression has been described to indicate left main or proximal left anterior descending coronary artery stenosis, with previous guidelines suggesting to treat as a ST-elevation myocardial infarction equivalent.^1^ However, recent data suggests that only 10% will have a culprit lesion with these ECG findings, but approximately 60% will have severe coronary artery disease.^2^ Although the ECG pattern may not always correspond with a culprit lesion, it should raise concerns for significant coronary artery disease, and an urgent cardiology consultation is needed. History such as APS, which is a multisystem autoimmune disease associated with coronary artery disease, should also increase suspicion for acute myocardial infarction especially in those who are less than 45 years of age.^3^

**CPC-EM Capsule**

What do we already know about this clinical entity?

*Evidence is mixed on whether ST-segment elevation in aVR with diffuse ST-segment depression represents a ST-elevation myocardial infarction equivalent*.

What is the major impact of the image(s)?

*These findings can represent significant coronary artery disease, with this case showing an isolated 99% stenosis in the left main coronary artery*.

How might this improve emergency medicine practice?

*These electrocardiogram findings should raise concerns for acute coronary syndrome and urgent consultation with cardiology to improve outcomes*.
